# Bullying Victimization in Young Females with Fragile-X-Syndrome

**DOI:** 10.3390/genes11091069

**Published:** 2020-09-11

**Authors:** Lorena Joga-Elvira, Carlos Jacas, María-Luisa Joga, Ana Roche-Martínez, Carme Brun-Gasca

**Affiliations:** 1 Consorcio Corporación Sanitaria Parc Tauli, Sabadell, 08208 Barcelona, Spain; ana.roche.mar@gmail.com (A.R.-M.); carme.brun@uab.cat (C.B.-G.); 2 Departamento de Psiquiatría y Medicina Legal, Universitat Autònoma de Barcelona, Bellaterra, 08193 Barcelona, Spain; 3 Servicio de Psiquiatría, Hospital Universitari Vall d’Hebron, 08035 Barcelona, Spain; cjacas1@gmail.com; 4 Departament de Psicología Clínica i de la Salut, Universitat Autònoma de Barcelona, Bellaterra, 08193 Barcelona, Spain; 5 Hospital San Joan de Deu, 08950 Barcelona, Spain; mjoga@sjdhospitalbarcelona.org

**Keywords:** fragile X syndrome, young females, bullying, behavior

## Abstract

The aim of this study is to investigate the risk associated with girls with fragile X syndrome (FXS) suffering bullying in the role of a victim and its effects on their adaptive behavior, socialization style, and emotional state. A neuropsychological assessment was carried out on a sample of 40 participants (26 FXS positive and 14 control group) using the following instruments: WISC-V, SENA, BAS-2, ABAS-II. The results show that the group of girls with FXS presented higher ratios of lack of social support and isolation from classmates. This finding suggests that problems with social interaction and communication in the group of girls with FXS could lead to difficulties in interpreting social signals and identifying situations of bullying correctly, placing them in a very vulnerable situation.

## 1. Introduction

Different definitions of bullying can be found in the scientific literature. In a recent study by Hellström and Beckman [1], bullying was defined as repetitive aggressive behaviors with harmful or hurtful intent and including some form of power imbalance between those involved [2]. It can take the form of direct verbal or physical aggression, relational aggression such as rumor spreading, or gossiping, either online or offline [3]. Bullying can be either direct or indirect. Direct bullying consists of physical and verbal forms of intentional negative behaviors. Indirect or relational bullying comprises exclusion or social isolation, lying, talking behind ones’ back, spreading rumors, or manipulating relationships [4].

Three main roles with the following characteristics have been defined within bullying: bully or perpetrator (easily frustrated, has positive attitude toward violence, impulsive, sees threats where none exist); victim (insecure, may believe that he or she deserves to be teased/taunted and harassed, perceived as weak or different, socially isolated, unassertive); and bully-victim (prone to irritating others and creating tension socially, quick tempered and emotionally reactive, reacts to being bullied with provocation, fighting back, and then may claim self-defense) [5].

The 2019 UNESCO report [6] warns that school violence and bullying are serious global problems among the general population. It is estimated that 15.4% of school children in Spain have suffered bullying. The Save the Children in Spain report finds that 39.65% of children have been involved in a situation of bullying during their childhood. Of these, 27.43% have experienced bullying between one and three times. Regarding the consequences of having been involved in bullying, numerous studies have reported that it can have a significant impact on mental health (anxiety and depression symptomatology, risk of suicide, suicide ideation), quality of life (sleeping problems), risky behavior, and reduced academic performance and chance of future employment [7,8,9,10].

Regarding the population with intellectual disabilities (ID), Maïano et al. [4] found that youths with ID are involved in bullying more frequently not only as victims (36.3%) but also as perpetrators (15.1%) and perpetrator-victims (25.2%). The study reported that this victimization can take different forms: verbal (50.2%), physical (33.3%), relational (37.4%), or cyber (38.3%). Their results showed that the perpetrator prevalence ratio in subjects with ID is the same as that of children with normal development. However, the victim and perpetrator-victim ratios were higher in the group with ID than in the group with normal development. Social exclusion and a lack of social support are the most frequent forms of bullying suffered by subjects with different types of disabilities or special needs [4,11]. Children with disabilities are more victimized by their peers than their classmates without disabilities and they are slower in developing social skills, which can increase their vulnerability to bullying [12].

Another study exploring the relationship between a psychiatric diagnosis and the fact of suffering bullying in patients aged 12 revealed that 25.4% of the sample group had reported having suffered from bullying. The frequencies of bullying found depending on the diagnosis were: 63.3% ID, 62.5% Autism Spectrum Disorder (ASD), 39.4% attention deficit hyperactivity disorder (ADHD), and 25% learning disabilities. Higher ratios of bullying are reported in subjects with a diagnosis of ID or ASD irrespective of their gender [13].

Fragile X syndrome (FXS) is a genetic condition associated with an alteration of the *FMR1* gene (fragile X mental retardation 1), which leads to the total absence or partial reduction of the FMRP protein. One of the main areas of expression of the *FMR1* gene is the brain. FMRP plays an important role in synaptic plasticity, axonal-dentritic development and learning and memory processes [14]. Epidemiological studies show an estimated frequency of 1.4:10.000 males and 0.9:10.000 females [15] and a prevalence of 1 per 2.500 to 7.000 males and 1 per 2.500 to 11.000 females [16].

FXS is the leading cause of hereditary ID. In females, it has been described that 50% of subjects present a variable degree of ID (between mild and moderate) and the remaining 50% present an intelligence quotient (IQ) in the medium range [17]. Girls with FXS present higher levels of social anxiety, social avoidance, and shyness, and they tend to isolate socially, have poor eye contact, and show difficulties in establishing an adequate rapport with others, ASD traits (social interaction and communication deficits, stereotypical movements), learning difficulties and depression [18].

The review by Maïano et al. [4] identified the following risk factors of suffering bullying in the population with special needs: physical appearance (characteristic physical traits); poor academic performance and inappropriate behavior; limited social relations and unstable friendships; deficits in social skills and problem solving. Considering the previously described characteristics of girls with FXS, this group would be expected to be at higher risk of suffering bullying during school years, but as far as the authors of this paper know there are no publications to this effect. Hence, the aim of the present work is to study the risk of girls with FXS suffering bullying in the role of victim and its effect on their capacity of adaptation, style of socialization, and emotional state compared with the control group (CG) of a similar age and IQ.

The study was designed based on two hypotheses:

(1) In our sample, at least 50% of girls with an FXS diagnosis have suffered some type of bullying in the role of victim.
(2) There will be an association between suffering bullying in the role of victim and their adaptive behavior, style of socialization, and emotional state. These associations will be different depending on the presence of FXS diagnosis.

## 2. Materials and Methods

The present research was carried out at Sabadell’s Parc Taulí Hospital, which belongs to the Clinical Experience Units Network for minority diseases (Barcelona, Spain) and is also a member of the Fragile X Syndrome Clinical Research Consortium. It follows on from a previous study in which the relationship between linguistic functions and executive function was assessed using other cognitive and behavioral variables (social perception, quantitative reasoning, and adaptive behavior). It was approved by the Ethics Committee of the same hospital (reference 2016595).

The Spanish FXS Federation, and different associations of patients with FXS (Catalonia, the Basque Country and Valencia) or other genetic diseases (D’Genes Murcia) took part in disseminating the project through professional social networks such as LinkedIn and by emailing psychopedagogy consultants and special education colleges in the area.

Informed consent was obtained from the participants’ legal guardians and participants gave their verbal consent.

### 2.1. Participants

It has been described that girls with FXS present a great variability in terms of IQ: approximately 50% of women with FXS have some kind of intellectual disability (ID) to varying degrees (between limit and moderate), while the other 50% have a general cognitive ability that sits within the midrange [17]. This is a fact that we find in our daily clinical practice, and hence the sample features participants with IQ ranging from intellectual disability to within midrange, which is a realistic representation of the way FXS manifest in girls. To avoid any potential confounding, a statistical analysis was performed to ensure that there were no significant differences between both groups in terms of IQ, age, socio economic status (SES), and ADHD symptoms. The means comparison was made using the Student’s *t*-test, and no statistically significant differences were found between groups in these variables (Table 1).

A total of 40 girls aged between 7 and 16 took part in the study. They were divided into the following two groups:

(1) FXS group: *n* = 26. Participants recruited through the Parc Taulí Hospital and the different patient associations.
(2) Control group (CG): *n* = 14. Participants were recruited through outpatients’ appointments at the Neuropediatrics Unit of Sabadell’s Parc Taulí Hospital and standard and special education schools in the area.

The inclusion and exclusion criteria were as follows:

- Inclusion criteria:

- FXS group: female, aged between 7 and 16 years, FXS confirmed by genetic study (>200 repetitions).
- Control Group: female, aged between 7 and 16 years.

- Exclusion criteria:

- FXS criteria: presence of comorbid ASD or ADHD, absence of expressive language, acquired neurological disorders (epilepsy, head injury).
- Control Group: meeting the diagnostic criteria for ASD or ADHD, absence of expressive language, acquired neurological disorders (epilepsy, head injury) and other cognitive-behavioral disorders. Participants without ID did not undergo a genetic study for ethical reasons, but subjects were questioned explicitly about their family history of FXS and the presence of clinical symptoms compatible with an FXS diagnosis.
- In both groups, participants who meet all DSM-5 criteria for the diagnosis of ASD and ADHD were excluded from the sample. Participants who only showed some symptoms were not excluded. The assessment was performed by an expert in pediatric neurology, psychology, or psychiatry.

### 2.2. Measures and Procedure

The assessment sessions for the participants from the Barcelona Metropolitan Area took place in Sabadell’s Parc Taulí Hospital. The lead investigator travelled to the autonomous regions where the rest of the participants lived to carry out the assessments, thus ensuring as little inconvenience as possible for the participants and their families. The participating autonomous communities were Catalonia, Madrid, the Basque Country, Galicia, Valencia, and Murcia.

First, a document explaining the project was produced and sent to the patient associations for dissemination among the families. When families showed interest in taking part in the study, the lead investigator would travel to the pertinent autonomous region on previously agreed days to carry out the assessments in the facilities provided by the associations. The assessment was divided into three separate one-hour sessions to ensure that participants did not become overtired. Last, parents were asked to complete the corresponding questionnaires. Due to the general and reading comprehension difficulties shown by most of the subjects, administration of self-reported questionnaires was not possible.

#### 2.2.1. Measures and Scales

- Wechsler Intelligence Scale for Children-Fifth Edition (WISC-V) Spanish version: a measure to assess participants’ IQ ($\bar{x}$ = 100 ± 15) [19].
- Adaptative Behavior Assessment System-Second Edition (ABAS-II) Spanish version: parents’ version. A measure to assess adaptive behavior in the conceptual domain (communication, functional, and self-directed academic skills), the social domain (recreation and social), the practical domain (self-care, home life, use of community resources, and health and safety), and the global domain ($\bar{x}$ = 100 ± 15) [20].
- Child and Adolescent Evaluation System (SENA): parents’ version. An assessment of a wide range of emotional and behavioral problems (depression, anxiety, hyperactivity and impulsivity, defiant behavior, substance abuse, eating disorders, learning difficulties, among others), contextual problems (problems with the family, at school, and with peers), areas of vulnerability (problems regulating emotions, isolation, rigidity, and so on), and psychological resources (self-esteem, social competence and integration, emotional intelligence, and so on). For these behavioral measures, standard scores were obtained ($\bar{x}$ = 50 ± 10). The critical items “Risk of school bullying”, “Lack of social support”, “Isolated by classmates”, “Insult by classmates” and “Afraid of a classmate” were used to establish the presence or absence of bullying. For critical items, the outcomes are binary variables (presence vs. absence) [21].
- BAS-2. Battery of socialization: parents’ version. Estimation scales in four domains facilitating socialization (leadership, joviality, social sensitiveness, and respect-self-control), three domains that disrupt socialization (aggressiveness-obstinacy, apathy-withdrawnness, and anxiety-shyness), and a global social adaptation or criterial-socialization scale (percentile scores) [22].

#### 2.2.2. Statistical Analysis

The statistical analysis was carried out using the SPSS Statistics for Windows Version 25.0 software. The Student’s *t*-test was used to compare the control variables age and IQ for the two groups.

For the binary variables, corresponding contingency tables were built to assess the risk of suffering bullying in the FXS group compared with the control group, and the Odds Ratio (OR) was calculated with its confidence intervals at 95%. For the quantitative variables, the effect of presenting FXS on emotional state, adaptive behavior, and socialization style was investigated using linear regression models that included an interaction period between the group and the presence of bullying.

## 3. Results

In our sample, regarding IQ, 50% of the participants in the FXS group scored in the range of mild/moderate ID, while the remaining 50% scored in the medium/medium-low range. Of the latter, 69.2% obtained scores corresponding to the borderline range and 30.7% to the medium range. On comparing the means using the Student’s *t*-test, no statistically significant differences were found between the control variables age and IQ (Table 1). The Cronbach’s Alfa values are summarized in Table 2.

Taken as a whole, it was found that 50% of the girls in the FXS group presented the condition “Risk of school bullying” versus 42.9% of the CG; 42.3% of the FXS group presented “Lack of social support” versus 28.6% of the CG; 38.5% of the girls with FXS presented “Isolated by classmates” versus 28.6% of the CG; 23.1% of the FXS group presented “Afraid of a classmate” versus 14.3% of the CG; and 26.9% of the girls with FXS were “Insulted by their classmates” versus 35.7% of the CG. The results show that the girls with FXS presented a higher ratio of lack of social support and isolation by their classmates than the girls in the CG (13.7% and 9.9% more, respectively). Conversely, the girls with CG presented a higher ratio of insulted by their classmates than the girls with FXS (8.8% more). Despite the trend found, the differences between groups were not statistically significant. (Table 3).

Regarding the multivariant analysis of the effect of FXS on joviality, social competence and integration, depression, personal resources, and self-care with respect to the control group, it was observed that there was a statistically significant interaction between some of the components of Bullying (“Risk of school bullying”, “Lack of social support”, “Isolated by classmates”, “Insulted by classmates” and “Afraid of a classmate”) and the presence of the syndrome.

**Figure 1:**
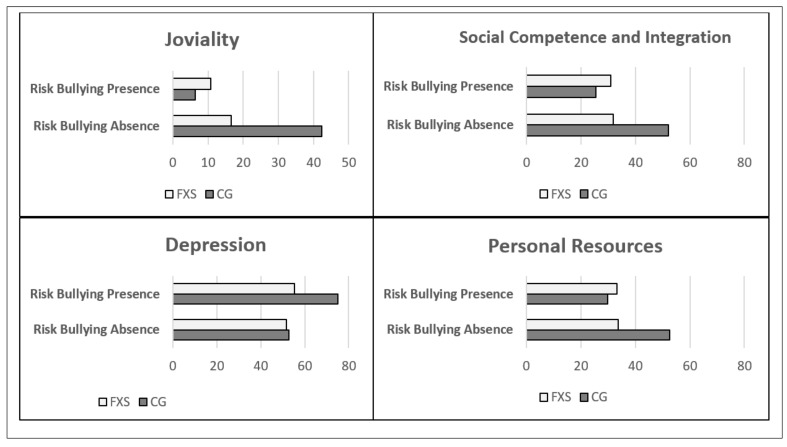
Interaction of “Risk of bullying” with Group on Joviality, Social Competence and Integration, Depression, and Personal Resources. FXS = Fragile X Syndrome. CG = control group.

On analyzing the data on the presence of “Risk of school bullying”, a significantly different effect was observed depending on the group on joviality (*p* = 0.012), social competence and integration (*p* = 0.001), depression (*p* = 0.035), and personal resources *(p* = 0.004). To this effect, the girls in the CG whose parents’ scores had indicated “Risk of school bullying” presented significant worsened average scores in the variables joviality (absence $\bar{x}$ = 42.38 ± 28.75 versus presence $\bar{x}$ = 6.34 ± 7.53 *p* = 0.009), social competence and integration absence $\bar{x}$ = 52.13 ± 7.68 versus presence $\bar{x}$ = 25.34 ± 14.95 *p* = 0.005), personal resources (absence $\bar{x}$ = 52.5 ± 5.66 versus presence $\bar{x}$ = 29.83 ± 12.19 *p* = 0.004), and depression (absence $\bar{x}$ = 52.75 ± 9.98 versus presence $\bar{x}$ = 75.00 ± 18.29 *p* = 0.03), compared with their classmates whose scores did not reflect “Risk of School Bullying”. Conversely, in the group of girls with FXS, no significant differences were observed in the means of those who presented “Risk of school bullying” and those who did not in the variables joviality (absence $\bar{x}$ = 16.59 ± 15.46 versus presence $\bar{x}$ = 10.70 ± 10.66 *p* = 0.285), social competence and integration (absence $\bar{x}$ = 31.85 ± 11.52 versus presence $\bar{x}$ = 31.01 ± 9.75 *p* = 0.842), personal resources (absence $\bar{x}$ = 33.69 ± 10.09 versus presence $\bar{x}$ = 33.31 ± 13.24 *p* = 0.934), and depression (absence $\bar{x}$ = 51.69 ± 12.83 versus presence $\bar{x}$ = 55.46 ± 10.86 *p* = 0.427). On comparing the two groups, it was found that in the absence of “Risk of school bullying”, the participants with FXS started with a lower score in all the variables (joviality, social competence and integration, and personal resources), except in the case of depression, in which the two groups start with similar scores (Figure 1 and Table 4).

**Figure 2:**
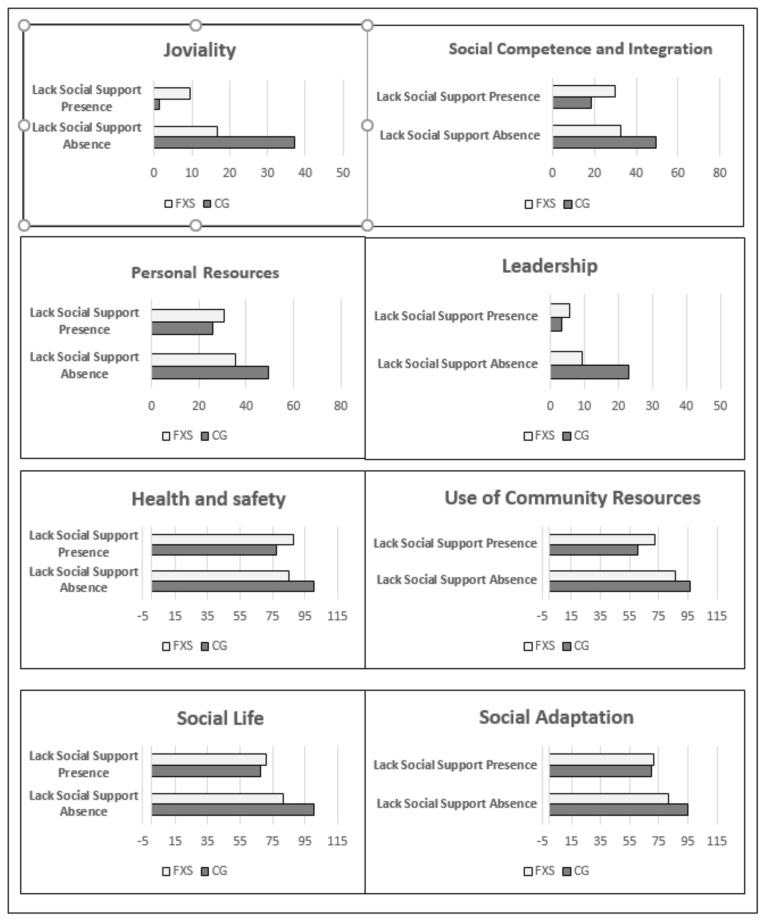
Interaction of “Lack of Social Support” with Group on Joviality, Integration and Social Competence, Personal Resources, Leadership, Health and security, Social Life, and Social Adaptation. FXS = Fragile X Syndrome. CG = control group.

Regarding “Lack of social support”, the effect on joviality (*p* = 0.03), social competence and integration (*p* = 0.001), and personal resources (*p* = 0.021) was found to differ depending on the group. As in the previous case, compared with their peers who did not suffer a “Lack of social support”, the CG participants whose parents’ scores indicated a “Lack of social support” presented significantly worsened average scores in the variables joviality (absence $\bar{x}$ = 37.10 ± 27.69 versus presence $\bar{x}$ = 1.5 ± 0.577 *p* = 0.003), social competence and integration (absence $\bar{x}$ = 49.50 ± 9.19 versus presence $\bar{x}$ = 18.50 ± 12.71 *p* = 0.01), and personal resources (absence $\bar{x}$ = 49.50 ± 8.22 versus presence $\bar{x}$ = 26.00 ± 13.44 *p* = 0.032). Conversely, in the group of girls with FXS, no significant differences were observed in the means of the variables joviality (absence $\bar{x}$ = 16.71 ± 14.20 versus presence $\bar{x}$ = 9.45 ± 11.23 *p* = 0.167), social competence and integration (absence $\bar{x}$ = 32.53 ± 11.51 versus presence $\bar{x}$ = 29.91 ± 9.17 *p* = 0.524), and personal resources (absence $\bar{x}$ = 35.47 ± 10.41 versus presence $\bar{x}$ = 30.82 ± 12.93 *p* = 0.339). On comparing the two groups, it was found that in the absence of “Lack of social support” the participants with FXS started with a lower score in joviality, social competence and integration, and personal resources. The same relational pattern, bordering on statistical significance, was found with the variables, leadership ability (*p* = 0.069), health and Safety (*p* = 0.077), use of community resources (*p* = 0.081), and social adaptation (*p* = 0.088) (Table 5). The lack of significance in the interaction between these variables could be due to a lack of strength in the test given the small size of the sample (Figure 2 and Table 5 and Table 6).

**Figure 3:**
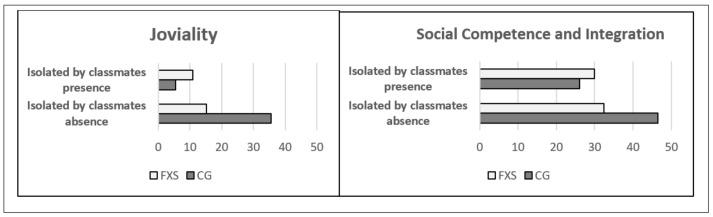
Interaction of “Isolated by classmates” with Group on Social Competence and Integration, and Joviality. FXS = Fragile X Syndrome. CG = control group.

Regarding the variable “Isolated by classmates”, just one significantly different effect was found depending on the group, social competence and integration (*p* = 0.049), and another on the borderline of significance, joviality (*p* = 0.063). Like in the previous cases, in the presence of being isolated by classmates the subjects in the CG had worse means compared with their peers who were not isolated by their classmates in social competence and integration (absence $\bar{x}$ = 46.50 ± 16.87 versus presence $\bar{x}$ = 26.00 ± 8.76 *p* = 0.013) and joviality (absence $\bar{x}$ = 35.50 ± 29.39 versus presence $\bar{x}$ = 5.50 ± 7.68 *p* = 0.012). Among the subjects in the FXS group, no significant difference was found in the means of the variables social competence and integration (absence $\bar{x}$ =32.38 ± 11.08 versus presence $\bar{x}$ = 29.90 ± 9.78 *p* = 0.557) and “joviality” (absence $\bar{x}$ = 15.27 ± 14.35 versus presence $\bar{x}$ = 10.90 ± 11.60 *p* = 0.411). Comparing the two groups, it was found that in the absence of being isolated by classmates the participants with FXS started with a lower score in “social competence and integration” and “joviality” (Figure 3 and Table 7).

**Figure 4:**
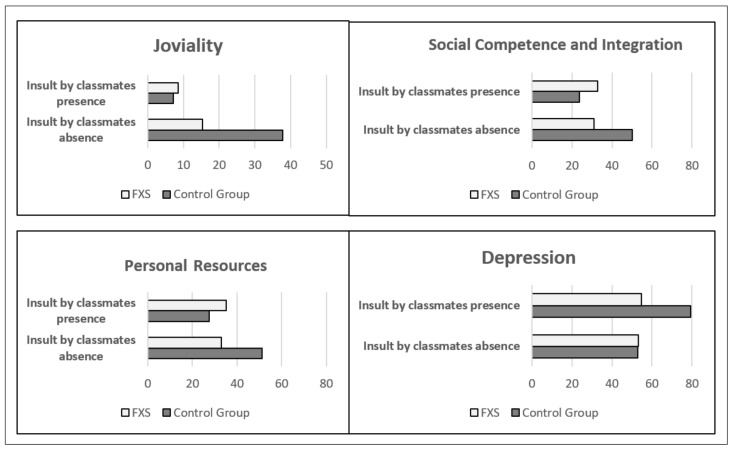
Interaction of “Insult by Classmates” with Group on Joviality, Social Competence and Integration, Personal Resources, and Depression. FXS = Fragile X Syndrome. CG = control group.

Regarding the variable “Insulted by classmates”, significantly different interactions were found depending on the group in social competence and integration (*p* = 0.001), depression (*p* = 0.006), and personal resources (*p* = 0.002), and borderline significant interactions in joviality (*p* = 0.072) were found. In the presence of being insulted by classmates, the subjects in the CG had worse means compared with their peers who were not insulted by their classmates in social competence and integration (absence $\bar{x}$ = 50.11 ± 9.39 versus presence $\bar{x}$ = 23.60 ± 16.02 *p* = 0.016), depression (absence $\bar{x}$ = 52.78 ± 9.34 versus presence $\bar{x}$ = 79.40 ± 16.52 *p* = 0.018), personal resources (absence $\bar{x}$ = 51.22 ± 6.53 versus presence $\bar{x}$ = 27.60 ± 12.18 *p* = 0.009), and joviality (absence $\bar{x}$ = 37.89 ± 30.07 versus presence $\bar{x}$ = 7.20 ± 8.07 *p* = 0.017). Among the subjects in the FXS group, no significant difference was found in the means of the variables social competence and integration (absence $\bar{x}$ = 30.89 ± 11.11 versus presence $\bar{x}$ = 32.86 ± 9.10 *p* = 0.654), depression (absence $\bar{x}$ = 53.16 ± 12.47 versus presence $\bar{x}$ = 54.71 ± 10.56 *p* = 0.757), personal resources (absence $\bar{x}$ = 32.89 ± 10.37 versus presence $\bar{x}$ = 35.14 ± 15.08 *p* = 0.725), and joviality (absence $\bar{x}$ = 15.39 ± 14.44 versus presence $\bar{x}$ = 8.71 ± 8.54 *p* = 0.171). In the absence of being insulted by classmates, the girls in the FXS groups started with a lower score in social competence and integration, personal resources and joviality, but in the case of depression both groups started with a similar score (Figure 4 and Table 8).

**Figure 5:**
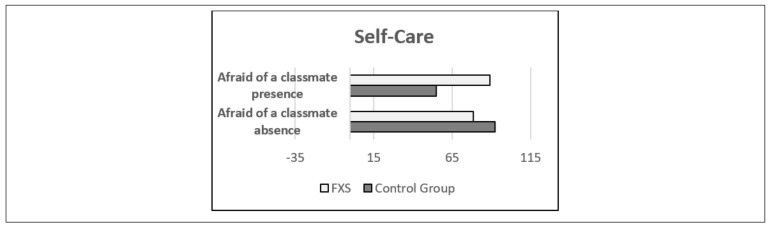
Interaction “Afraid of a classmate” with Group on Self-care with Group. FXS = Fragile X Syndrome. CG = control group.

Lastly, regarding the variable “Afraid of a classmate”, just one significantly different effect was found depending on group, in self-care (*p* = 0.014). To this effect, the girls in the CG who were afraid of a classmate presented worse means compared to their peers who were not afraid of a classmate in self-care (absence $\bar{x}$ = 92.50 ± 26.24 versus presence $\bar{x}$ = 55.00 ± 0 *p* < 0.001). No significant difference in means was found in the group of girls with FXS in self-care (absence $\bar{x}$ = 78.42 ± 17.24 versus presence $\bar{x}$ = 89.17 ± 20.35 *p* = 0.279). In the absence of being afraid of a classmate, the participants in the FXS group started with a lower score in social competence and integration, personal resources, joviality, and self-care, while the two groups started with a similar score in depression (Figure 5 and Table 9).

## 4. Discussion

The aim of the present work was to study the ratio shown by girls with Fragile X who had suffered bullying in the role of victim and the effect this has had on their adaptive behavior, socialization style, and emotional state.

First, it was observed that although the ratio of the girls with FXS who had suffered bullying at school according to their parents’ assessment was similar to that of the girls in the control group, there was discrepancy between the two groups in the type of bullying suffered. Among the girls with FXS, a higher ratio was found in lack of social support and isolation by classmates. These findings coincide with results previously published in the scientific literature, which identify social exclusion as the form of bulling most frequently suffered by people with some type of disability (physical, intellectual or social) or special needs [4,11,12]. Conversely, the girls in the CG presented a higher ratio of insults from classmates.

Second, it was found that the girls who had suffered bullying showed more symptoms of depression, lower levels of joviality and personal resources, and more difficulties with self-care and social competence and interaction. On analyzing the influence of the components of bullying on each of the variables studied, it was observed that the risk of suffering bullying was related to worsening levels of joviality, personal resources, social competence and integration, and depressive symptoms; lack of social support was associated with lower levels joviality, personal resources and social competence and integration; isolation by classmates negatively affected the subjects’ social competence and integration; suffering insult from classmates negatively affected their personal resources, depressive symptoms, and social competence and integration; and being afraid of a classmate was associated with lower levels of self-care.

In all the interactions among the group and the different forms of bullying, a similar pattern was observed: in the presence of different forms of bullying, the girls in the CG showed a more pronounced worsening than the girls in the FXS group. The girls with FXS tended to present ASD traits such as deficits in social communication and interactions, stereotypic behavior, and poor eye contact [18]. To this effect, difficulties in social communication and interaction could lead to difficulties in interpreting social signals and correctly identifying situations of bullying, which would mean a lowered awareness of the situation and, therefore, a decreased effect on their levels of joviality, personal resources, self-care, and social competence and integration in comparison with the girls in the control group. It is important to note that presenting difficulties in identifying situations of bullying does not imply the absence of it. This fact could place girls with FXS in a hugely vulnerable situation given that faced with a situation of bullying they would not be able to correctly identify the signals and, therefore, would not be able to adequately manage and resolve the situation. This finding concurs with results previously reported in the literature by different authors, who point out that girls with disabilities can present a lack of or a delay in developing social skills, which increases their vulnerability to bullying [12,23,24].

### Strenghts and Limitations

One of the limitations of this study is the relatively small size of the sample. However, despite having only 26 girls with FXS participating in the study, it is the largest sample of girls of child-juvenile age collected to date in Spain, and one of the largest at an international level. The person responsible for carrying out the investigations travelled to different parts of the country over a period of four years to reach the highest possible number of participants. Other limitations were the great difficulty we had in finding a large number of girls for the CG, and the fact that groups are very heterogeneous. This could somehow bias the results. Nonetheless, there were no significant differences between the groups in terms of the control variables, age and IQ. Furthermore, although the girls with ID in the control group had undergone a genetic analysis which had ruled out FXS, the girls without ID did not undergo a genetic study for ethical reasons. However, the participants were evaluated clinically to assess whether they presented symptoms compatible with FXS (physical traits, cognitive-behavioral characteristics), and they were asked specifically about any previous family history of FXS. Lastly, it must be remembered that the presence of bullying was ascertained using questionnaires administered by participants’ parents. In future research, this information should be extended by adding data from assessments made by both teachers and the girls themselves.

## 5. Conclusions

Last, it must be noted that the present findings could indicate, that when planning interventions for girls with FXS in situations of bullying, apart from mediating and intervening in the immediate situation, there must be a more specific focus on direct work with the girls themselves, placing special emphasis on developing their social skills, and on mitigating any deficits in their social communication and interaction.

## Figures and Tables

**Table 1:** Descriptive measures and distribution of age, IQ, ADHD Symptoms, and socioeconomical status.

Variables	FXS Group *n* = 26	Control Group *n* = 14	Significance
**Age** **M (SD)**	10.58 (3.384)	10.50 (2.345)	0.940
**IQ** **M (SD)**	71.50 (14.795)	76.36 (16.80)	0.351
**ADHD symptoms** **M (SD)**	81.54 (4.341)	68.29 (6.984)	0.104
**Socioeconomical status**			
**Low**	3.8%	16.7%	0.211
**Medium**	88.5%	75%	
**High**	7.7%	8.3%	

FXS = Fragile X Syndrome. IQ = intelligence quotient. ADHD = Attention Deficit Hyperactivity Disorder. M = mean. SD = standard deviation. M = mean.

**Table 2:** Adaptative Behavior Assessment System- Second Edition (ABAS-II) Cronbach’s Alfa.

	Cronbach’s	Alfa
	Control Group	Fragile X Syndrome
**Social adaptation**	0.778	0.691
**Conceptual Adaptation**	0.755	0.832
**Practical Adaptation**	0.842	0.903

**Table 3:** Odds ratio of bullying depending on the group.

	FXS	CG	Odds Ratio	Significance	95% Confidence Interval	
					Inferior	Superior
Risk of school bullying	50%	42.9%	1.333	0.666	0.360	4.933
Lack of social support	42.3%	28.6%	1.833	0.392	0.454	7.408
Insult by classmates	26.9%	35.7%	0.663	0.563	0.164	2.676
Afraid of a classmate	23.1%	14.3%	1.8	0.507	0.312	10.39
Isolated by classmates	38.5%	28.6%	1.563	0.532	0.384	6.356

FXS = Fragile X Syndrome. CG = control group.

**Table 4:** Interaction of “Risk of bullying” with Group on Joviality, Social Competence and Integration, Depression, and Personal Resources (central tendency and variability).

Variables	β	Confidence Interval 95%		Significance
		Inferior	Superior	
**Joviality**				
Risk of Bullying	−36.042	−54.630	−17.453	<0.001
Group	−25.792	−41.502	−10.081	0.002
Interaction	30.151	7.012	53.289	0.012
**Social Competence and Integration**				
Risk of Bullying	−26.792	−38.715	−14.869	<0.001
Group	−20.279	−30.199	−10.358	<0.001
Interaction	25.946	11.210	40.681	0.001
**Depression**				
Risk of Bullying	22.250	8.396	36.104	0.002
Group	−1.058	−12.585	10.469	0.853
Interaction	−18.481	−35.603	−1.359	0.035
**Personal Resources**				
Risk of Bullying	−22.667	−34.627	−10.707	<0.001
Group	−18.808	−28.759	−8.856	<0.001
Interaction	22.282	7.501	37.036	0.004

**Table 5:** Linear regression model with Interaction of “Lack of Social Support” with Group on “leadership”, “Health and security”, “Use of community resources”, “Social adaptation”.

	CG “Lack of Social Support”		*Significance*	FXS “Lack of Social Support”		*Significance*
	Absence	Presence		Absence	Presence	
**Leadership**	$\bar{x}$ = 23.10 ± 20.44	$\bar{x}$ = 3.25 ± 2.87	0.014	$\bar{x}$ = 9.43 ± 7.18	$\bar{x}$ = 5.64 ± 7.39	0.211
**Health and safety**	$\bar{x}$ = 100.50 ± 15.54	$\bar{x}$ = 77.5 ± 18.48	0.03	$\bar{x}$= 85.36 ± 23.57	$\bar{x}$ = 87.37± 16.94	0.773
**Social adaptation**	$\bar{x}\;$ = 95.10 ± 11.0	$\bar{x}$ = 69.75 ± 13.20	0.021	$\bar{x}$ = 81.57 ± 14.89	$\bar{x}$ = 71.6 ± 14.06	0.093
**Use of community resources**	$\bar{x}$ = 96.50 ± 15.46	$\bar{x}$ = 61.25 ± 7.50	0.001	$\bar{x}$ = 86.43 ± 19.36	$\bar{x}$ = 72.27 ± 14.89	0.05

FXS = Fragile X Syndrome. CG = control group.

**Table 6:** Interaction of “Lack of Social Support” with Group on Joviality, Integration and Social Competence, Personal Resources, Leadership, Health and security, Social Life, and Social Adaptation. FXS = Fragile X Syndrome. CG = control group (central tendency and variability).

Variables	β	Confidence Interval 95%		Significance
		Inferior	Superior	
**Joviality**				
Lack of Social Support	−35.600	−56.683	−14.517	0.002
Group	−20.386	−35.141	−5.630	0.008
Interaction	28.340	2.832	53.849	0.03
**Social Competence and Integration**				
Lack of Social Support	−31.000	−43.556	−18.444	<0.001
Group	−16.967	−25.631	−8.302	<0.001
Interaction	28.376	13.256	43.496	0.001
**Personal Resources**				
Lack of Social Support	−23.500	−36.674	−10.326	0.001
Group	−14.033	−23.124	−4.943	0.003
Interaction	18.852	2.987	34.716	0.021
**Leadership**				
Lack of Social Support	−19.850	−34.207	−5.493	0.008
Group	−13.671	−23.719	−3.623	0.009
Interaction	16.058	−1.313	33.429	0.069
**Health and Safety**				
Lack of Social Support	−23.000	−46.403	0.403	0.054
Group	−15.143	−31.521	1.236	0.069
Interaction	25.370	−2.944	53.685	0.077
**Use Community Resources**				
Lack of Social Support	−35.250	−54.944	−15.944	0.001
Group	−10.071	−23.854	3.711	0.147
Interaction	21.094	−2.733	44.922	0.081
**Social Adaptation**				
Lack of Social Support	−25.350	−41.694	−9.006	0.003
Group	−13.529	−24.967	−2.090	0.022
Interaction	15.142	−4.632	34.917	0.088

**Table 7:** Interaction of “Isolated by classmates” with Group on Social Competence and Integration, and Joviality. FXS= Fragile X Syndrome. CG = control group (central tendency and variability).

Variables	β	Confidence Interval 95%		Significance
		Inferior	Superior	
**Joviality**				
Isolated by Classmates	−30.000	−52.281	−7.719	0.010
Group	−20.233	−35.608	−4.858	0.011
Interaction	25.633	−1.437	52.704	0.063
**Social Competence and Integration**				
Isolated by Classmates	−20.500	−35.321	−5.679	0.008
Group	−14.125	−24.224	−4.026	0.007
Interaction	18.025	0.090	35.960	0.049

**Table 8:** Interaction of “Insult by Classmates” with Group on Joviality, Social Competence and Integration, Personal Resources, and Depression. FXS= Fragile X Syndrome. CG = control group (central tendency and variability).

Variables	β	Confidence Interval 95%		Significance
		Inferior	Superior	
**Joviality**				
Insult by Classmates	−30.689	−51.196	−10.182	0.004
Group	−22.500	−37.510	−7.490	0.004
Interaction	24.014	−2.230	50.258	0.072
**Social Competence and Integration**				
Insult by Classmates	−26.511	−39.092	−13.930	<0.001
Group	−19.216	−28.343	−10.089	<0.001
Interaction	28.474	12.420	44.528	0.001
**Depression**				
Insult by Classmates	26.622	12.950	40.294	<0.001
Group	0.380	−9.539	10.299	0.9539
Interaction	−25.066	−42.512	−7.619	0.006
**Personal Resources**				
Insult by Classmates	−23.622	−35.892	−11.352	<0.001
Group	−18.327	−27.229	−9.426	<0.001
Interaction	25.870	10.213	41.528	0.002

**Table 9:** Interaction “Afraid of a classmate” with Group on Self-care with Group. FXS= Fragile X Syndrome. CG = control group (central tendency and variability).

Variables	β	Confidence Interval 95%		Significance
		Inferior	Superior	
**Self-care**				
Afraid of a classmate	−37.500	−69.597	−5.403	0.023
Group	−14.079	−29.575	1.417	0.074
Interaction	48.246	10.595	85.896	0.014
